# Mentorship in advanced endoscopy: a conversation with Peter Draganov and Dennis Yang

**DOI:** 10.1016/j.igie.2025.03.006

**Published:** 2025-04-03

**Authors:** Peter Draganov, Dennis Yang, Vivek Kaul

**Affiliations:** 1 Division of Gastroenterology and Hepatology, University of Florida, Gainesville, Florida, USA; 2 Center for Interventional Endoscopy, Advent Health, Orlando, Florida, USA; 3 Division of Gastroenterology and Hepatology, University of Rochester Medical Center, Rochester, New York, USA

## Editor’s Introduction

Endoscopic training and practice have evolved rapidly in recent times.^1^, ^2^, ^3^ In this series, we seek out and highlight unique personal and professional attributes of highly successful mentor–mentee dyads who share key aspects of their journey and provide useful insights to peers. In this discussion, I had the opportunity to participate in a 3-way recorded conversation with Drs Peter Draganov and Dennis Yang (Video 1, available at www.igiejournal.org), each of whom has made significant contributions to advanced endoscopy in general, and third-space endoscopy in particular. We explored several key aspects of training and professional development as they reflected on their careers and milestones. Hopefully, current and emerging mentors and future mentees will find these pearls of wisdom useful for their own careers.

Vivek Kaul, MD, FASGE

*iGIE* Associate Editor for iNSIGHTS

**Vivek Kaul (VK): What attracted you to endoscopy?**

**Peter Draganov** (**PD):** Both my parents are physicians, so I’ve been immersed in medicine since I was born. Medicine as a profession came very naturally to me. In medical school, I was very much attracted to specialties that were “fixing things” using your hands in one way or another. At that time, gastrointestinal (GI) endoscopy was mostly diagnostic, so early on it was not one of my primary choices, but the cognitive aspect of gastroenterology attracted me. Over the years, endoscopy has evolved significantly, and it has ultimately combined the cognitive aspect of medicine with the technical aspect of endoscopy. When I chose to pursue gastroenterology, it was a great disappointment to my father, who was a cardiologist, but it has been a great choice for me!

**Dennis Yang** (**DY):** I was really drawn to the physiology and pathophysiology of the GI tract. I felt like it was one of the specialties that made more sense to me from a cognitive aspect and, like Dr Draganov mentioned, having the ability to perform endoscopy and experience immediate therapeutic results is certainly one of the most gratifying aspects of what we do.

**VK: Who were your early mentors?**

**PD:** I came to the United States immediately after medical school, and I ended up at the Medical University of South Carolina for my internal medicine residency. Peter Cotton and Rob Hawes joined that institution at the exact same time, and the rest is history. At the time, it was one of the leading programs in the world for advanced endoscopy, with multiple international people coming to train. If you look at who’s who in gastroenterology currently, a vast majority of people have come through that program. Although both were my mentors, they have very different styles. Rob was very straightforward, easy to communicate with, so you knew exactly what was going on. Peter, even now, is very difficult to know exactly when he’s very happy or mad at me! He is consistent in his communication without many ups and downs, just slow and steady as they say.

**DY:** I didn’t know I wanted to do advanced endoscopy until I was in the first year of a general gastroenterology fellowship. Early on when I was a resident at the Mayo Clinic in Rochester, I had the opportunity to work with Dr David Alquist, may he rest in peace. He was one of the brightest and kindest people I have ever met, and he really took me under his wing. I really didn’t know how to establish myself and develop a career in gastroenterology at that time. He introduced me to research and allowed me to work in his laboratory, so I’m always very grateful for his patience and his commitment to my growth despite him being a very busy physician. I wanted to come to Florida for gastroenterology fellowship, and he recommended University of Florida as the premier program in the state. He mentioned several names, including Dr Draganov.

**VK:** **Dennis, you hit upon several key principles: a great mentor will demonstrate kindness, the ability to take somebody under their wing, and then to guide them dispassionately. Most of us have our own biases and our own prejudices in life, but when it comes to mentorship in the professional realm, we have to advise according to what we think is in the best interest of the mentee.**

**DY:** I still remember I started my first month as a general gastroenterology fellow in the outpatient clinic. I think they were a bit concerned that I was coming from Mayo Clinic Rochester and would be overwhelmed by the high volume of patients, so they said, “Let’s ease him in with outpatient clinic,” and I was fortunate to be paired up with Dr Draganov. That was a great opportunity for me to start interacting with him early on and see his panel of patients, which were mainly pancreaticobiliary and advanced endoscopy referrals. That initial exposure started getting me into the field, I think.

**VK: Peter, what were some of the qualities that made Dennis an attractive candidate for gastroenterology fellowship from your perspective?**

**PD:** Dennis came to us through the general gastroenterology match and then stayed on for advanced endoscopy training. His background was stellar on paper. When looking at potential candidates, certainly a solid educational background, personality, and commitment to education and learning are important attributes. Dennis was very committed to academics. Eventually, when you get to work with somebody, people fall into different categories: problem solvers and problem creators. Dennis is a problem solver so when there is an issue, he tries to resolve it and, in most cases, successfully so. To me, that was one of the most significant attributes.

**VK: Dennis, can you share any advice you have on how an advanced endoscopy candidate can best prepare himself/herself for the fellowship interview process?**

**DY:** First and foremost, remember why you became a physician in the first place, which is to take care of patients. As a trainee, sometimes people get so bogged down about what they need to do for that application that they forget this essential fact. If you keep that as your primary goal, it tends to reflect in how you carry yourself, how you perform, and in the way you engage with colleagues. Often, this results in building a good reputation as a trainee, which in turn helps generate excellent letters of recommendations, which for me is the most important thing that I look for in any applicant’s package. Second, given the competitiveness in the field, involvement in scholarly activity is necessary, mainly because we are looking for people who we believe are going to push the field forward, and that tends to be through research and education. This is where being able to find a mentor early on who may be able to guide you through this process is indispensable.

**VK: It really becomes critical to also have a plan for what you are going to do with your training. There is oversaturation of talent in some geographic areas while there is a great need in other regions, but I think people gravitate to communities and areas that they feel most attracted to. One of the things that we talk about to trainees is where are you going to go, what your platform will be, and what are you going to do with this specialized training**.^1^

**PD:** This is a hot topic of discussion at the American Society for Gastrointestinal Endoscopy: do we have too many advanced endoscopists or not enough? The reality is that we don’t know because in the United States, we have decentralized medicine, and it’s hard to know how many people exactly do a meaningful volume of endoscopic retrograde cholangiopancreatography (ERCP), for example. My personal biased opinion is that we have enough; it’s simply an issue of distribution. In the desirable markets, we have oversaturation but, at the same time, there are not enough in geographic areas of need. You cannot easily fix that, particularly in the United States, where medical care/access is not driven only by governmental mandate. In Bulgaria where I grew up, medical school was free. After you graduate, for 3 years you go to a destination that they assign you to, usually underserved areas. That is not feasible in this country so I think our focus should be to continue to train good physicians and then allow the market dynamics to do its job.

**VK: Thinking about the current state of advanced endoscopy training, what changes do you think are necessary to ensure fellows become highly skilled in specific procedures while maintaining a comprehensive understanding of the field?**

**PD:** We are at a crossroads. When I was in medical school, ERCP was the only advanced procedure, and then when I was getting into my gastroenterology fellowship, endoscopic ultrasound (EUS) was emerging. Now we do deep enteroscopy, place metal stents in the luminal GI tract, perform ablation with various techniques, drain pancreatic pseudocysts, do necrosectomies, perform endoscopic mucosal resection on lesions that previously went to surgery, and not to even mention third-space endoscopy with per-oral endoscopic myotomy, endoscopic submucosal dissection, and full-thickness resection. All of these are now routine procedures that we do daily. Regarding training, it’s very difficult to lump all this into a 1-year additional training program. For this reason, we extended our training program to 18 months. From day one, we start with third-space endoscopy so everything moves in parallel. However, this is still suboptimal. I have a list of things that I will do when I become a King! One of the things on my list is to fix subspecialty training. I would say a good model will be to do 2 years of general gastroenterology fellowship, and then your third year should be focused on your area of interest, might that be inflammatory bowel disease, hepatology, etc. Then, if you want to add a fourth year, that could be utilized for advanced endoscopy so you don’t graduate fellowship about the same time when you start getting your Social Security checks.

**DY:** It’s kind of a (good) problem that we created: the sub-specialization of advanced endoscopy now has provided so many specialty procedures that it’s nearly impossible to try to learn them all during 1 year of advanced endoscopy fellowship. I tell fellows when they interview that the goal is not to train somebody who’s a “jack-of-all-trades” because then you’re not going to be good at anything. I still think that, currently as it stands, the 1-year advanced endoscopy fellowship should focus on ERCP and EUS. An advanced fellow’s goal should still be to become a robust endoscopist and be able to treat the majority of pancreaticobiliary disease. Certainly, most of our programs nowadays provide some exposure to sub-subspecialty endoscopy, such as endobariatrics, endohepatology, interventional EUS, and/or third-space endoscopy, but the comprehensive cognitive understanding of how to incorporate these procedures into patient care and the technical competence to perform these procedures takes time and dedicated mentorship.

**VK: Should research be a part of advanced endoscopy training?**

**PD:** Yes. If nothing else, people will read research manuscripts for the rest of their career. If you’re not writing one, you must be able to grasp details about methodology, and the best way to learn it is by doing a project or 2. My first publication was a case report, and my gosh it took a lot of time and effort to publish that case report. It was a very positive experience and gave me perspective into what it takes to get published so I think the fellows need to be involved in research, but that doesn’t mean that they must spend much of their time on research.

**DY:** Most of the research we do as advanced endoscopists is clinical research so it goes back to how we can improve patient care. I think incorporating that into fellowship training is important because it stimulates our trainees to think of ways to move the field forward and how to provide better patient care. It’s very rewarding to me to have trainees who share that same passion that you do in terms of thinking of new ways to solve a problem. That is what drives me to work every day. One of the most important things is to understand the process, the logistics of carrying out a project once you have a question in mind. Educating trainees during the process will serve them a long way during their career.

**VK: Dennis, what was your time like as a trainee with Dr Draganov?**

**DY:** I truly enjoyed working with Dr Draganov. I appreciate how he approaches patient care. His approach is pragmatic, and providing the best patient care was always his primary goal. I also always felt that he had my best interest in mind. He was genuinely invested in my career growth. An important piece of advice I have for mentees is you need to understand that mentors take time out of their busy schedule for you. You need to be grateful for this and cherish that unique opportunity that you have been provided.

**VK: Any major dos and don’ts for mentors?**

**PD:** You must be genuinely invested in that person. I would discourage not being straightforward and not being open about sharing your thoughts. I think it’s very important whether you like something or you don’t like something. We live in an era where feedback is difficult to give and difficult to receive, but it is very important to communicate with the mentees in real time as the training proceeds.

**DY:** One of the things I keep telling myself is to understand the timeline that the mentee has with you. I try not to get them involved in potential projects that they may not see to completion and which may not translate into something positive and productive for the mentee. For the mentee, the very first time I meet them I tell them I’m going to be fully committed to you. That means I’m going to spend my time meeting with you on a regular basis, but I expect the same type of commitment from you.

**VK: Mentors sometimes have some relative insecurity when trainees stay in the region after training. Peter, you seem to still be very busy despite having trained so many people, some of whom may have stayed regionally. Any thoughts on how a mentor should approach that delicate issue that arises in many communities nationally?**

**PD:** I personally have zero concerns about that and that is because I believe all boats rise with the tide.

**VK:** **Dennis, you set up shop in Florida not too far away from the Peter. You too have major world-class competition in your local market as well. Tell us what mentees should be thinking when they enter a highly competitive market.**

**DY:** Florida is a very big state with many people (and growing), and I think that wherever you go there’s always going to be plenty of opportunities to take care of patients. I learned from Dr Draganov and others who have influenced me throughout my career to focus on myself and try to be the best version of yourself, do a good job, and the rest falls into place.

**VK: How do you balance a complex professional life with your personal life?**

**PD:** I recently read an article that said it’s not work–life balance, it’s work–life integration. If you’re talking about work–life balance, you’re doing one thing and that means you’re compromising the other thing, so you must look at it as integration. I have tried everything. I go 1 week on vacation and don’t touch e-mail, and oh my gosh! I come back, and it’s a disaster! So instead, I try to integrate work and personal life and make them work the best I can both ways. This concept of integration, rather than balance, is what has helped me.

**DY:** I like this new concept of integration. I’ve made strides over the years. I used to spend most weekends in my office working on research. Once I had kids, I would work in research on the weekends early in the morning and then spend time with them later. Now that they’re toddlers, I’ve tried to become more and more efficient. I wake up a little bit earlier to do work and then spend time with my family when they are awake to not disturb the balance.

**VK: What are some key factors to consider when navigating different practice environments such as private practice or academic institutions?**

**DY:** There are going to be differences in a non-university health system setting when it comes to academic pursuits. From an efficiency standpoint, such a platform tends to be more fast-paced but being in a hybrid practice, where the institution operates outside a traditional university setting but still values academic pursuits, there is support for these activities. On the downside, obviously navigating the private practice aspect of medicine for someone who’s only been in academia previously takes some time, adjustment, and re-calibration.

**PD:** The skill sets are universally applicable. The navigation of the systems is quite different, of course. In some cases, you need a different personality skill set to navigate academics versus private practice, but some of the problems that we face are universal no matter where you are so you have to be nimble and adjust to whatever environment you are in.

**VK: Do the 2 of you still collaborate on projects sometimes?**

**DY:** People always ask me about this. Oftentimes, after I moved here to Advent Health, it just felt like an extension from where I was, so not much has changed in terms of what I do with Dr Draganov except I don’t have him in the endoscopy unit (with me) every day. From a collaborative standpoint, everything has been the same, and I would say in many ways even more productive because now we have the benefit of having 2 institutions rather than just 1 extending our reach.

**Figure 1:**
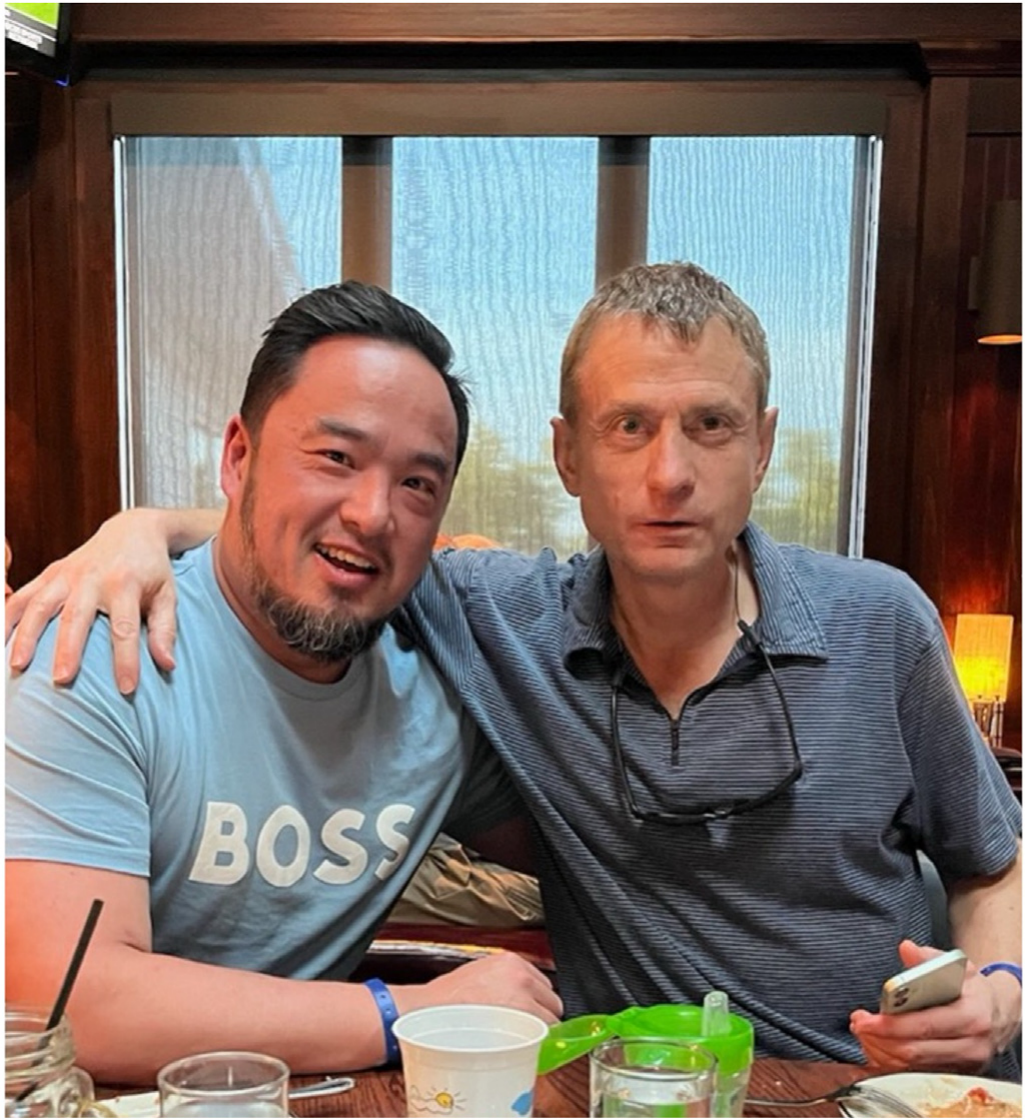
Drs Yang and Draganov visiting at home.

**Figure 2:**
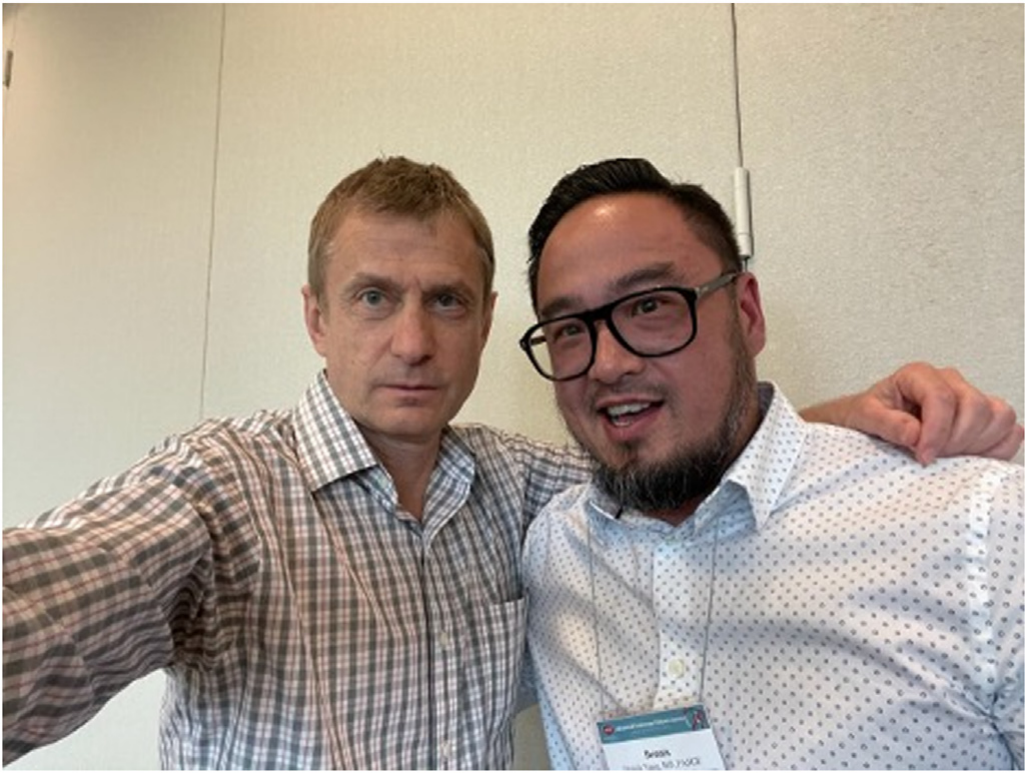
Drs Draganov and Yang at a conference.

**Figure 3:**
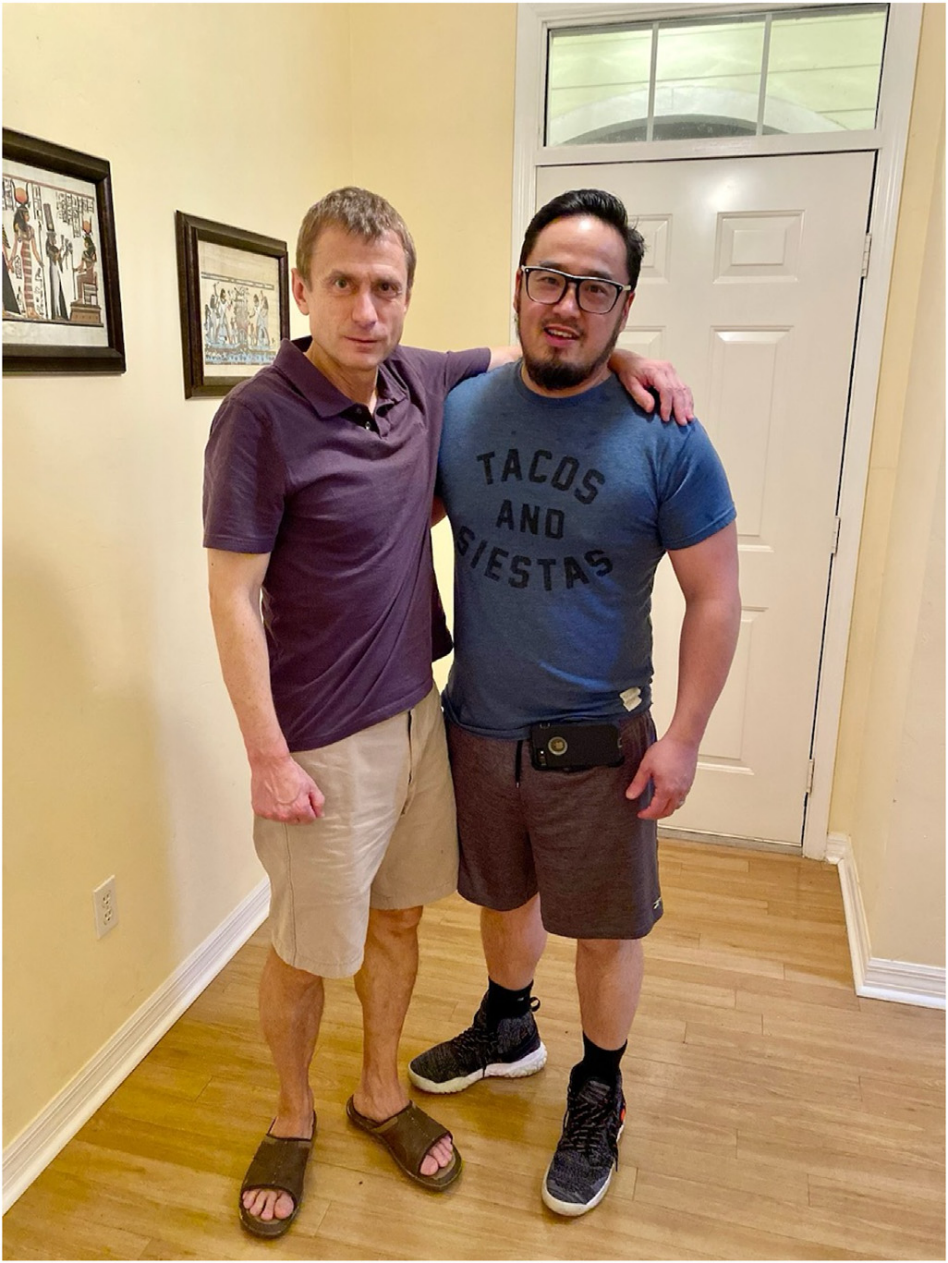
Drs Draganov and Yang socializing after work.

**PD:** If anything, things have continued to expand and to get better. We’re still relatively close geographically so we see each other with some frequency. Most importantly, we have similar values, and we also enjoy each other’s company (Figure 1, Figure 2, Figure 3).

**VK:** **These are lifelong relationships, and the mentorship continues whether it’s passive or active or unseen by the rest of the world, so that’s important. Last question, and I always ask this: what does the future hold for you?**

**PD:** I don’t know. We can plan only that much. I certainly am very much enjoying what I currently do but to quote a friend of mine, “if you’re not going up, you’re going down.” You must constantly work to find ways to do better. I think I’m doing a good job right now, but I also know I can do better.

**DY:** What drives me to work every day is to think of ways of making things better for our patients. That’s what keeps us on our toes. Never settle and just go through the motions of work every day. Think about every day as a challenge and an opportunity to improve, whether that’s yourself or your practice or your trainees and so forth.

## Disclosure

All authors disclosed no financial relationships.
